# The effect of negative attention bias on mobile phone addiction in sports majors: a moderated mediation model

**DOI:** 10.3389/fpsyg.2025.1645214

**Published:** 2025-11-19

**Authors:** Zheng Tian, Sheng Qu, Han Jing, Beile Cong, Yushan Li, Rui Qiu

**Affiliations:** 1 School of Physical Education and Sport Science, Fujian Normal University, Fuzhou, China; 2 Baoji University of Arts and Sciences Education College, Baoji, China; 3 Tangshan Normal University President’s Office, Tangshan, China; 4 Air Force Medical University Military Medical Psychology School, Xi’an, China

**Keywords:** negative attention bias, mobile phone addiction, depression, anxiety, moderated mediation model

## Abstract

**Objective:**

This study aimed to explore the intricate relationship between negative attention bias and mobile phone addiction, with a particular focus on the mediating role of depression and the moderating role of anxiety.

**Methods:**

Through an online questionnaire survey of 891 college students majoring in physical education, multiple validated scales were employed for measurement, and advanced statistical methods were utilized for data analysis.

**Results:**

The results revealed a significant positive correlation between negative attention bias and mobile phone addiction. Depression was found to partially mediate the relationship between the two, and anxiety significantly moderated the association between depression and mobile phone addiction.

**Conclusion:**

These findings not only enrich the theoretical research on mobile phone addiction, but also provide empirical evidence for the development of targeted intervention strategies, thus holding great significance for promoting the mental health of sports majors.

## Introduction

### The prevalence and significance of mobile phone addiction

In the contemporary digital epoch, mobile phones have become an integral and omnipresent part of people’s lives (Ko et al., 2021; Li et al., 2022). The pervasiveness and functionality of these devices have brought about significant changes in the way individuals communicate, access information, and engage in various aspects of daily living (Gui and Gerosa, 2021). However, the increasing prevalence of excessive mobile phone use has led to a growing concern known as mobile phone addiction (Loleska and Pop-Jordanova, 2021). This phenomenon has evolved into a salient issue that transcends age cohorts, exerting its influence not only on adolescents and young adults but also on individuals across diverse life stages (Wang et al., 2024).

Students majoring in physical education may demonstrate distinct psychological characteristics and behavioral tendencies relative to their peers in other academic disciplines, attributable to the specific nature of their professional training environment (e.g., high-intensity training, competitive evaluations), curricular demands, and career development trajectories (Zhou et al., 2021). This provides a unique perspective for studying the complex relationships between negative attention bias, depression, anxiety, and mobile phone addiction.

### Negative attention bias as a cognitive precursor

The current study sought to examine the complex association between negative attention bias and mobile phone addiction, emphasizing the mediating effect of depression and the moderating influence of anxiety. By focusing on college students majoring in sports, this research aims to address existing gaps in the literature and offer empirical support for the development of targeted mental health interventions tailored to this particular demographic.

Negative attention bias, a concept well-established within cognitive psychology, denotes the pronounced propensity of individuals to preferentially perceive and allocate their attention to negative stimuli over positive stimuli (Ito et al., 1998).

The direct impact of negative attention bias on mobile phone addiction demands immediate attention. Although existing research has provided some indications of a possible connection, the precise nature and strength of this direct link remain elusive (Shi et al., 2025).

Extensive research in the field of psychology has demonstrated a strong association between negative attention bias and several psychological disorders, with depression and anxiety being among the most prominent (Veerapa et al., 2020).

### The mediating role of depression

Depression, characterized by a persistent state of low mood, loss of interest and pleasure, self-blame, and a host of other symptoms, has been the subject of extensive research in the field of mental health (Kroenke et al., 2001).

Cognitive Bias Theory posits that college students majoring in sports, due to prolonged exposure to intensive training and frequent competitive evaluations, are predisposed to developing negative cognitive patterns related to professional setbacks, such as training injuries, competition failures, or critical feedback (Alvi et al., 2022; Leung et al., 2022). This predisposition manifests as a negative attentional bias, wherein these students involuntarily concentrate more on adverse professional information rather than positive stimuli. Such biased cognitive processing serves as a foundational mechanism for the onset of depressive symptoms, as sustained focus on negative experiences undermines mood and self-efficacy (Zuo et al., 2025).

Numerous studies have suggested a potential relationship between depression and mobile phone addiction (Feng et al., 2022; Višnjić et al., 2018). The intricate nature of this relationship continues to be a focus of active research, as the precise mechanisms and pathways by which depression may lead to mobile phone addiction have not been comprehensively elucidated.

Although a few studies have tentatively suggested an association between negative attention bias and mobile phone addiction via the conduit of depression, the exact mechanisms and pathways through which this mediation unfolds are far from being fully elucidated (Hou et al., 2021). Depression, with its complex symptomatology and far-reaching psychological implications, could potentially act as a catalyst, amplifying the impact of negative attention bias on mobile phone use (Wei et al., 2023). It might be that individuals experiencing depressive episodes are more inclined to retreat into the virtual world offered by their phones, using it as a means to numb emotional pain or fill a void of social interaction (Yi and Li, 2021). Conversely, excessive mobile phone use, perhaps leading to social isolation or a distorted self-perception, could trigger or exacerbate depressive symptoms, which in turn further fuel the cycle of phone addiction (Peper and Harvey, 2018).

According to Stress and Coping Theory, college students who major in sports encounter distinct professional stressors, including career uncertainty and competitive pressure. When depression arises from negative attentional bias, their rigorous training schedules and athletics-centered social environments restrict their access to adaptive coping strategies, such as face-to-face psychological support or diverse recreational activities. Consequently, mobile phone use emerges as a convenient but maladaptive coping mechanism, providing temporary relief from negative emotions or a compensatory sense of gratification (De-Sola et al., 2016; Liu et al., 2024; Spitzer et al., 2006).

### The moderating role of anxiety

Anxiety, characterized by intense worry, agitation, and a feeling of looming disaster, may serve as a significant influencing factor (Gao and Tien, 2025), either attenuating or intensifying the association between depression and mobile phone addiction (Kuru and çelenk, 2021). For example, among individuals exhibiting elevated anxiety levels, the compulsion to frequently check their phones for reassurance or diversion may be significantly intensified, thereby amplifying addictive behaviors (Ge et al., 2023). On the other hand, in some cases, anxiety could prompt individuals to seek healthier coping mechanisms, thereby mitigating the impact of depression on mobile phone use (Ge et al., 2023).

Based on the theoretical framework and existing research gaps, this study proposes the following specific hypotheses.

Hypothesis 1: Negative attention bias shows a significant positive association with mobile phone addiction among College Students Majoring in Physical Education. That is, the stronger the individual’s tendency to focus on negative stimuli, the higher the level of mobile phone addiction.

Hypothesis 2: Depression plays a mediating role in the relationship between negative attention bias and mobile phone addiction. Specifically, negative attention bias is expected to be positively associated with the level of depression, and depression is further hypothesized to show a positive association with mobile phone addiction.

Hypothesis 3: Anxiety moderates the mediating pathway of “negative attention bias → depression → mobile phone addiction,” specifically regulating the relationship between depression and mobile phone addiction. When anxiety levels are high, the positive association between depression and mobile phone addiction is expected to be stronger; conversely, when anxiety levels are low, the association is anticipated to be weaker.

**Figure 1:**
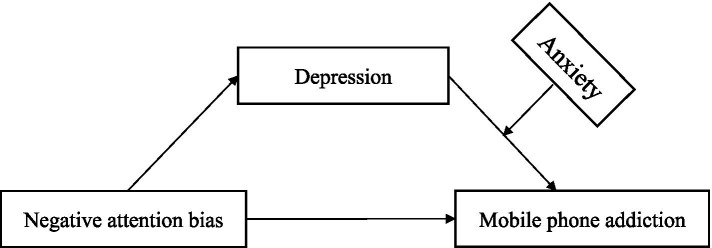
Theoretical model.

The theoretical model of this study is shown in Figure 1.

## Methods

### Participants

A convenience sampling method was employed to recruit undergraduate students majoring in physical education from the comprehensive population of universities in Shaanxi Province, Heilongjiang Province. An online questionnaire was distributed through department contacts, which yielded an initial pool of approximately 950 responses. After the exclusion of incomplete and insubstantial responses (e.g., those with patterned answering), a final sample of 891 questionnaires was retained for analysis, representing a response rate of approximately 89%. The sample size for this study was determined with reference to the sample size decision table proposed by Ahmad and Halim (2017). For a large population (typically exceeding 100,000), a representative sample size of approximately 384 is required at a 95% confidence level and a 5% margin of error. This study ultimately collected 891 valid questionnaires, well above this threshold, which ensures the sample’s representativeness of the population and the stability of the statistical analysis.

There were 734 males (82.4%) and 157 females (17.6%) aged between 19 and 24 years old, with an average age of 21.46 (SD = 1.56) years old. There were 7 people with junior high school education or below (0.8%), 377 people with senior high school education or junior college education (42.3%), 499 people with university education (56.0%), and 8 people with postgraduate education or above (0.9%). The sample comprised 324 only-child university students (36.4%) and 567 students with siblings (63.6%). The average time spent on mobile phones per day was 3.84 (SD = 3.36) hours.

### Informed consent

Prior to their inclusion in the study, all participants provided informed consent. They were fully informed about the nature and purpose of the research, the procedures involved, potential risks and benefits, and their rights as participants. Participants were assured that their participation was voluntary and they could withdraw from the study at any time without penalty. Confidentiality of participants' data was strictly maintained. The data collected were anonymized and used solely for the purposes of this research.

### Measures

#### Attention to positive and negative information scale

In this study, the Negative Information Attention Subscale (ANI) of the Positive and Negative Information Attention Scale (APNI) was used to assess individuals’ attention to negative information in life (Noguchi et al., 2006; Qin et al., 2015). A representative item is as follows: I always pay attention to past situations that made me feel terrible.

ANI consists of 10 items, with the following response scale; 1 (completely inconsistent) to 5 (completely consistent). The higher the score, the more obvious the negative attention bias. In this study, the Cronbach *α* of this subscale was 0.888.

#### Patient health questionnaire

In this study, the patient health questionnaire (PHQ-9) was used for screening and evaluation of depressive symptoms (Kroenke et al., 2001). A typical item is as follows: Having thoughts of being better off dead or hurting myself in some way. A total of 9 items were used, with scores ranging from 0 (not at all) to 3 (almost every day). The total score ranges from 0 to 27 points, where zero to four indicates no depression, five to nine indicates mild depression, ten to fourteen indicates moderate depression, fifteen to nineteen indicates moderate to severe depression, and twenty to twenty-seven indicates severe depression. The higher the score, the higher the degree of depression. The Chinese version of PHQ-9 has been verified with good reliability and validity (Cuidong et al., 2009). In this study, the Cronbach *α* of this scale was 0.957.

#### Smartphone Application-Based Addiction Scale

Smartphone Application-Based Addiction Scale (SABAS) was used to measure the level of mobile phone addiction. The scale was developed by Csibi in 2016 based on Griffiths ‘addiction model (Csibi et al., 2016; Griffiths, 2005). A characteristic example of an item as follows: Have I tried to reduce my use of social media but failed? It contains a total of 6 entries, each of which is graded on a Likert 6-point scale from “1 = strongly disagree” to “6 = strongly agree.” The higher the score on the scale, the more likely the person was to be diagnosed with social media disorder. In this study, the Cronbach *α* of this scale was 0.925.

#### Generalized anxiety disorder 7

In this study, GAD-7 was used to screen and evaluate generalized anxiety symptoms, with a total of 7 items (Spitzer et al., 2006). A representative instance of item as follows: if I think something unpleasant might happen, I become restless. Scores ranging from 0 (not at all) to 3 (almost every day) were used, with a total score ranging from 0 to 21 points, where zero to four points indicate no anxiety, five to nine points indicate mild anxiety, ten to fourteen points are classified as moderate anxiety, and fifteen to twenty-one points are classified as severe anxiety; the higher the score, the higher the anxiety level (Shan and Li, 2015). In this study, the Cronbach’s *α* coefficient of this scale was 0.952.

#### Statistical analysis

SPSS 29.0 statistical software was used to conduct the common method deviation test, descriptive statistics, correlation analysis, and other basic analyses, with statistical significance defined as a *p*-value <0.05. The mediation and moderation analyses were conducted using Model 4 and Model 14, respectively, of the PROCESS macro for SPSS (Version 4.2). The Bootstrap method with 5,000 resamples was employed to test the significance of the effects, with significance determined by a 95% bias-corrected confidence interval that does not contain zero. Control variables included age and gender. Age was mean-centered. Gender was coded as 0 = male, 1 = female, with male as the reference category.

## Results

### Common method deviation test

In this study, Harman single factor analysis was used to test whether there was a common method bias (Hao and Lirong, 2004). The results show that there are 12 factors with feature roots greater than 1, and the variance explained by the first factor is 24.61%. Since the value of the first factor is lower than the threshold of 40%. It indicates a low probability of common method bias.

### Correlation analysis of negative attention bias, depression and mobile phone addiction

Table 1 presents a correlational analysis of the major variables under study and finds that, the higher the score of negative attention bias, the higher the score of mobile phone addiction, depression and anxiety. At the same time, there were significant positive correlations among negative attention bias, mobile phone addiction, depression and anxiety (*p* < 0.01).

**Table 1:** The mean, standard deviation and correlation coefficient of each variable (*n* = 891).

Variable	*M*	*SD*	1	2	3	4
1. Mobile phone addiction	15.43	6.97	1			
2. Negative attention bias	30.81	7.56	0.42^**^	1		
3. Depression	13.02	5.35	0.52^**^	0.32^**^	1	
4. Anxiety	10.47	4.35	0.53^**^	0.35^**^	0.93^**^	1

**p < 0.01, ***p < 0.001.

### The mediating effect of depression on negative attention bias and mobile phone addiction

The premise of mediating effect test is that there is a pairwise significant correlation between independent variables, dependent variables and mediating variables (Zhonglin et al., 2005). This study finds significant correlations among the three variables of negative attention bias, depression and mobile phone addiction.

**Figure 2:**
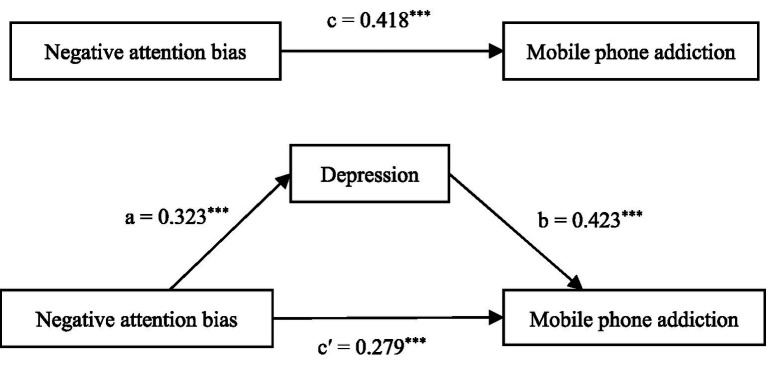
Mediating model of negative attention bias, depression and mobile phone addiction.

In this study, a step by step regression equation combined with Process was used to analyze the mediating effect. The main steps were as follows: First, negative attention bias was entered as the independent variable and mobile phone addiction as the dependent variable to test the association between negative attention bias and mobile phone addiction. Second, negative attention bias was used as the independent variable and depression as the dependent variable to examine the relationship of negative attention bias with depression. Finally, both negative attention bias and depression were included as independent variables in a model with mobile phone addiction as the dependent variable, to test their associations with mobile phone addiction. The specific results are shown in Table 2, Figure 2.

**Table 2:** Mediating effect of depression between negative attention bias and mobile phone addiction.

	Study variable	Implicit variable	*R* ^2^	Adjustment *R*^2^	*F*-value	*β*	*t*-value
1. Path *c*	Negative attention bias	Mobile phone addiction	0.175	0.174	188.677^***^	0.418	13.736^***^
2. Path *a*	Negative attention bias	Depression	0.105	0.104	103.837^***^	0.323	10.190^***^
3. Path *b*, *c*′	Depression	Mobile phone addiction	0.343	0.341	231.344^***^	0.423	15.040^***^
	Negative attention bias					0.279	9.687^***^

**p < 0.01, ***p < 0.001.

As shown in Table 2, Figure 2, negative attention bias was significantly positively associated with mobile phone addiction (*c* = 0.418, *p* < 0.001) and with depression (*a* = 0.323, *p* < 0.001). When both depression and negative attention bias were included in the regression model, depression was significantly positively associated with mobile phone addiction (*b* = 0.423, *p* < 0.001). Negative attention bias also remained significantly associated with mobile phone addiction (*c*’ = 0.279, *p* < 0.001). Based on these results, depression was found to mediate the relationship between negative attention bias and mobile phone addiction. The indirect effect (a*b) accounted for 32.69% of the total effect.

In order to further verify whether the mediating effect of depression is statistically significant, this study applied the simple mediating model of Process plug-in in SPSS and Bootstrap method to test the mediating effect of depression between negative attention bias and mobile phone addiction. With negative attention bias as the independent variable, mobile phone addiction as the dependent variable, and depression as the mediating variable, Model 4 was used in the Process plug-in with a sample size of 5,000 and a 95% confidence interval was evaluated. If the confidence interval did not contain 0, the mediating effect was significant. The results are shown in Table 3.

**Table 3:** Bootstrap test on the mediating effect of depression between negative attention bias and mobile phone addiction.

	Effect	BootSE	BootLLCI	BootULCI	Efficacy as a percentage
Indirect effect	0.129	0.023	0.086	0.175	33.44%
Direct effect	0.257	0.036	0.186	0.328	66.56%
Total effect	0.386	0.034	0.318	0.451	

As shown in Table 3, the indirect effect of negative attention bias on mobile phone addiction through depression is 0.129, with 95% confidence interval of [0.086, 0.175]. As the confidence interval does not include zero, the mediating role of depression is statistically significant. Therefore, these results support the hypothesis that depression serves as a significant mediator in the relationship between negative attention bias and mobile phone addiction.

### The moderating effect of anxiety on depression and mobile phone addiction

PROCESS model 14 was used to test the moderating effect of anxiety, and all variables were standardized before analysis. The results showed that the interaction term between anxiety and depression was significantly associated with mobile phone addiction (*β* = −0.021, *SE* = 0.005, *p* < 0.001). This suggests that anxiety moderates the relationship between depression and mobile phone addiction. The results are shown in Table 4.

**Table 4:** An examination of the moderating effects of anxiety.

Regression equation		Overall fit index			Significance of regression coefficients	
Outcome variable	Study variable	*R*	*R* ^2^	*F*	*β*	*t*
Mobile phone addiction	Negative attention bias	0.602	0.362	125.606^***^	0.237	8.979^***^
	Depression				0.381	3.979^***^
	Anxiety				0.430	3.744^***^
	Depression * Anxiety				−0.021	−3.909^***^

Each continuous variable in the model was standardized and then brought into the regression equation.

**p < 0.01, ***p < 0.001.

**Figure 3:**
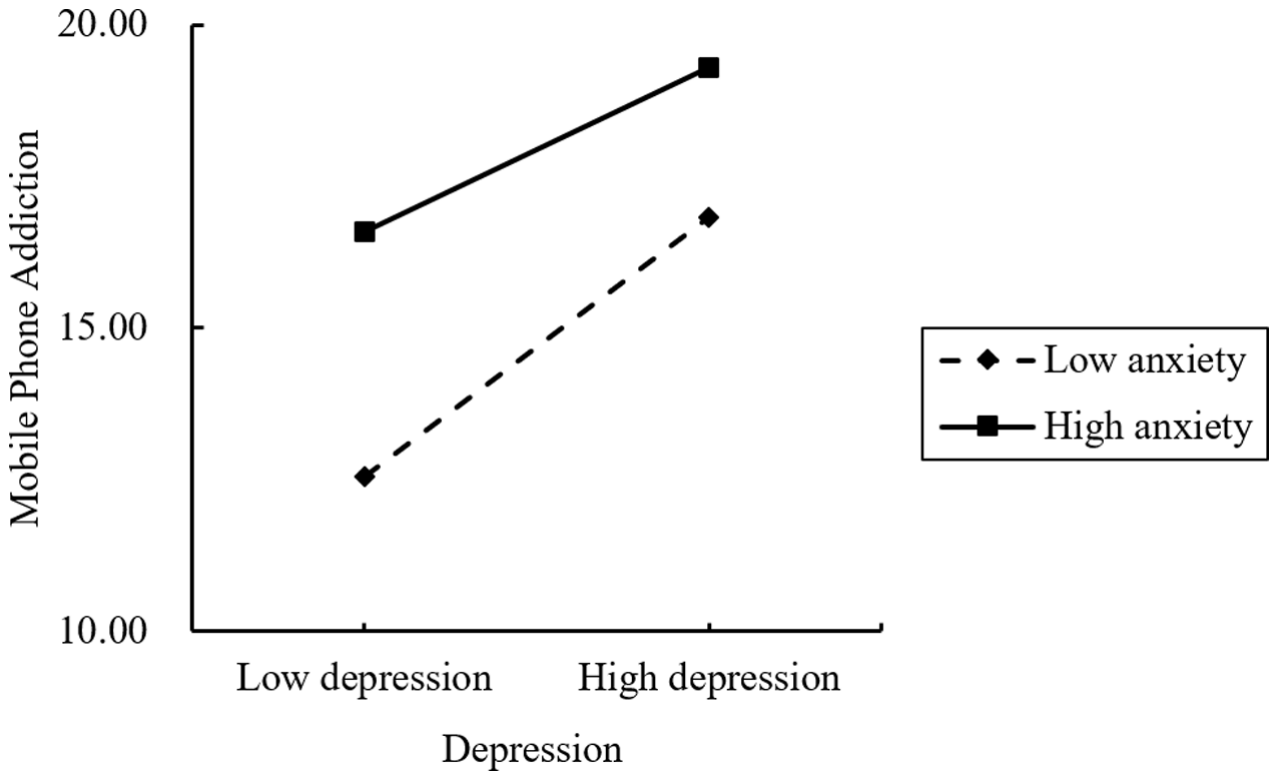
The influence of depression on mobile phone addiction at different anxiety levels.

In order to more clearly explain the nature of the interaction between depression and anxiety, depression and anxiety were divided into high and low levels according to 1 standard deviation each, and a simple slope test was performed, as shown in Figure 3. When the anxiety level was higher (M + 1SD), the association between depression and mobile phone addiction was weaker (*β* = 0.290, *t* = 3.152, *p* = 0.002). When anxiety levels were low (M − 1SD), the positive relationship between depression and mobile phone addiction was significantly stronger (*β* = 0.454, *t* = 4.425, *p* < 0.001).

## Discussion

The present study aimed to investigate the complex interplay between negative attention bias, depression, anxiety, and mobile phone addiction among undergraduate students specializing in physical education. The findings not only corroborate and extend existing literature in the field but also provide unique insights into this specific population, whose daily experiences are characterized by distinct physical, psychological, and social demands inherent to athletic training and competition.

### The direct association between negative attention bias and mobile phone addiction

Our results confirmed that negative attention bias is significantly associated with mobile phone addiction, a finding consistent with broader cognitive-behavioral frameworks that describe the mechanisms related to addictive behaviors. For sports majors, this relationship is relevant because of environmental stressors: chronic exposure to high-intensity training protocols, frequent performance appraisals, and the pressures of competitive athletics (Hutchinson and De Lucia, 2025; Yang et al., 2021). Whether ruminating on unmet training objectives, perceived physical inadequacies, or competitive setbacks, this heightened focus on adverse experiences establishes a cycle wherein mobile phone use emerges as a habitual coping strategy (Liu et al., 2022). Athletes may resort to mobile devices to disengage from negative cognitions, seeking transient relief through social media interactions, digital entertainment, or other forms of online distraction (Desclouds and Durand-Bush, 2021).

### The mediating role of depression

Our analysis revealed that depression functions as a critical mediator in the relationship between negative attention bias and mobile phone addiction, clarifying the emotional pathway through which cognitive biases translate into addictive behaviors. Sports students exhibiting a stronger propensity for negative attention bias are more inclined to ruminate on adverse experiences—including suboptimal competitive performance, critical feedback from coaches, or uncertainties regarding athletic career trajectories (Jama et al., 2023; Michel-Kröhler et al., 2024). Such rumination may exacerbate depressive symptomatology, as the persistent focus on negative events erodes mood and self-efficacy (Bandura and Locke, 2003). Consequently, these depressive states may drive increased mobile phone use, as athletes seek to escape distressing emotions. Mobile devices offer immediate distractions, social connectivity, and a temporary reprieve from the demands of their athletic endeavors, rendering them a compelling coping mechanism. This mediating effect highlights the necessity of dual-target interventions: addressing cognitive biases in isolation may be insufficient to curtail mobile phone addiction (Brown et al., 2017; Gulliver et al., 2012; Reardon et al., 2019).

### The moderating role of anxiety

Our findings demonstrated that anxiety moderates the association between depression and mobile phone addiction, with the strength of this relationship amplified at higher levels of anxiety. This aligns with theoretical models positing anxiety as a potentiator of emotional distress, augmenting the drive to seek relief through addictive behaviors. For athletes, who frequently operate within high-stakes environments where performance anxiety is endemic, this interaction is particularly salient (Sarkar and Fletcher, 2014). The pressure to achieve competitive success, meet training benchmarks, and maintain optimal physical conditioning can heighten anxiety, which in turn intensifies the impact of depression on mobile phone use. In such contexts, the co-occurrence of depression and anxiety engenders a stronger impetus to utilize mobile phones for comfort or distraction (Hoffner and Lee, 2015).

### Limitations and future directions

This study has several limitations that should be acknowledged, along with corresponding avenues for methodological refinement to advance understanding in this area. First, the cross-sectional design constrains our capacity to establish definitive causal relationships among the variables. While our proposed model posits that negative attention bias influences mobile phone addiction via depression, with anxiety moderating this pathway, alternative causal configurations remain plausible. Second, the operationalization of negative attention bias in the current study relied exclusively on self-report measures, which may incompletely capture the implicit cognitive processes underlying attentional tendencies. While self-report scales effectively assess explicit awareness of attentional patterns, they may fail to detect automatic, unconscious orienting toward negative stimuli. Third, the study conceptualized sports majors as a monolithic group, potentially overlooking significant variations among subgroups. For example, athletes participating in individual sports (such as track and gymnastics) encounter stressors related to personal performance and self-reliance, whereas those engaged in team sports (such as basketball and soccer) face pressures stemming from group dynamics. These distinctions may influence the magnitude of the association between depression and mobile phone addiction (Ji et al., 2024). Targeted improvements to address these limitations include: (1) Implementing longitudinal or ecological momentary assessment (EMA) designs to capture real-time fluctuations in attention, mood, and phone use. EMA, in particular, would enable data collection in the naturalistic settings of athletes’ daily lives, capturing how experiences such as intense training sessions or competitive outcomes influence cognitive biases, emotional states, and mobile phone use. (2) Combining self-report scales with behavioral tasks to operationalize negative attention bias more comprehensively, ensuring the capture of both explicit and implicit dimensions. (3) Expanding sampling to include diverse geographic, institutional, and cultural contexts, enabling exploration of the generalizability of findings and identification of context-specific influencing factors. By addressing these areas, future research can build upon the present findings to develop more targeted, effective strategies for preventing and mitigating mobile phone addiction among sports majors, ultimately supporting their holistic well-being and athletic achievement.

## Conclusion

This study systematically examined the complex relationships between negative attention bias, depression, anxiety, and mobile phone addiction among college students majoring in physical education, with the goal of validating a moderated mediation model. The key findings and academic implications are summarized below. First, negative attention bias was found to have a significant positive relationship with mobile phone addiction in this population. After controlling for depression, the direct positive effect of negative attention bias on mobile phone addiction was still statistically significant. This finding is consistent with cognitive-behavioral frameworks, indicating that sports majors’ increased attention to negative stimuli may drive habitual mobile phone use as a coping strategy. Second, depression influenced the link between negative attention bias and mobile phone addiction. Negative attention bias was found to predict depression, which in turn predicted mobile phone addiction. This suggests that negative attention bias not only directly influences mobile phone addiction, but also indirectly by exacerbating depressive symptoms. Third, anxiety significantly influenced the progression from depression to mobile phone addiction. The positive relationship between depression and mobile phone addiction was stronger in students with low anxiety than in those with high anxiety. This suggests that high anxiety may trigger alternative emotional distress pathways, weakening depression’s unique contribution to addiction. In summary, this study elucidates the psychological mechanisms underlying mobile phone addiction among college students majoring in sports. Its findings not only enrich the empirical literature on mobile phone addiction but also lay an evidence-based groundwork for developing targeted mental health interventions and addiction prevention strategies—ultimately supporting the holistic well-being and athletic development of this unique group.

## Data Availability

The raw data supporting the conclusions of this article will be made available by the authors, without undue reservation.
